# Correction to “Anxiety‐related defensive behavioral responses in mice selectively bred for High and Low Activity”

**DOI:** 10.1111/gbb.12897

**Published:** 2024-05-12

Winona C. Booher, Lucy A. Hall, Aimee L. Thomas, et al. *Genes Brain Behav*. 2021;e12730.

In the above article, incorrect versions of Figures 4 and 5 were published in error. In both figures, the Low Activity (dotted line) should be on top and the High Activity (solid line) should be on the bottom.

**FIGURE 4:**
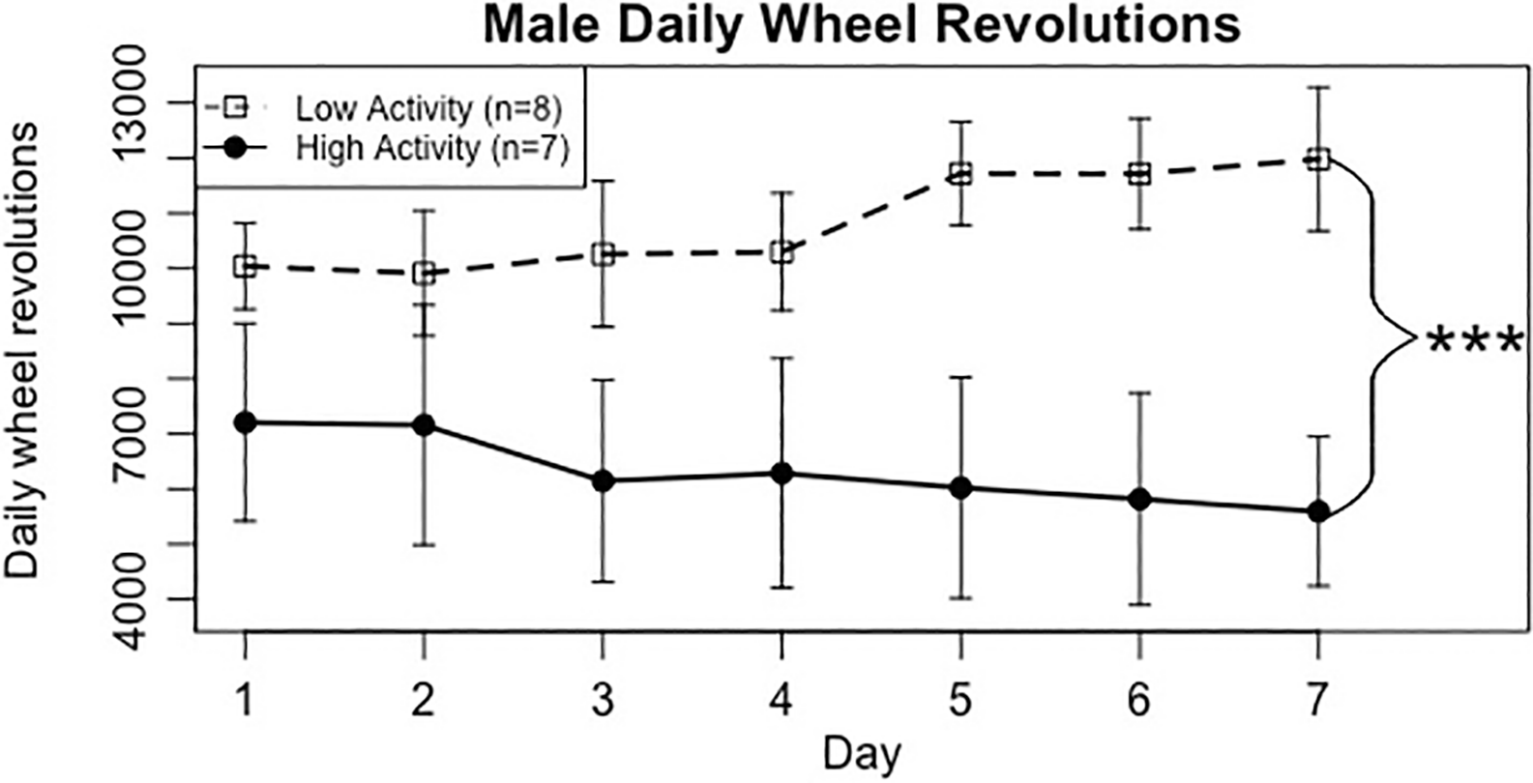
Average daily running wheel revolutions for male High and Low Activity mice. Data represent means ± standard error of the mean. Results were obtained using a two‐way repeated measures ANOVA, ***p < 0.001.

**FIGURE 5:**
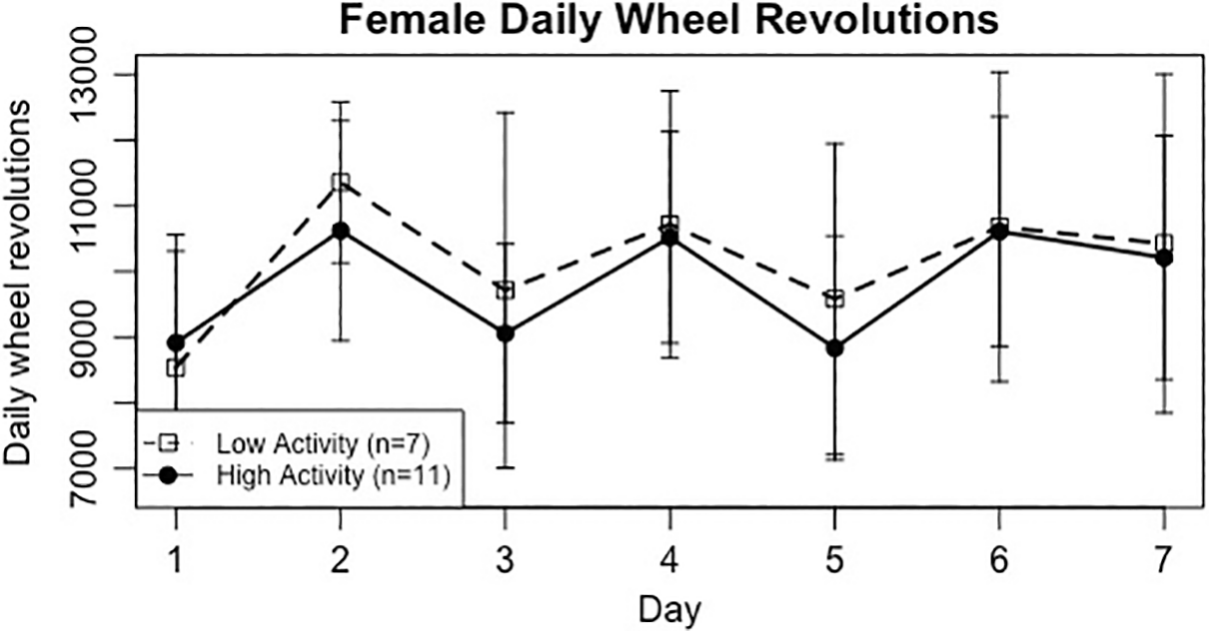
Average daily running wheel revolutions for female High and Low Activity mice. Data represent means ± standard error of the mean. Results were obtained using a two‐way repeated measures ANOVA.

The correct figures are reproduced below.

We apologize for this error.

